# Altered fear processing in adolescents with a history of severe childhood maltreatment: an fMRI study

**DOI:** 10.1017/S0033291716003585

**Published:** 2018-02-12

**Authors:** H. Hart, L. Lim, M. A. Mehta, A. Simmons, K. A. H. Mirza, K. Rubia

**Affiliations:** 1 Department of Child & Adolescent Psychiatry, Institute of Psychiatry, Psychology and Neuroscience, King's College London, UK; 2 Department of Neuroimaging, Institute of Psychiatry, Psychology and Neuroscience, King's College London, London, UK; 3 NIHR Biomedical Research Centre at South London and Maudsley Foundation NHS Trust and King's College London, Institute of Psychiatry, Psychology and Neuroscience, London, UK; 4 SLAM NHS Trust, Kennington, London, UK

**Keywords:** Child abuse, childhood maltreatment, fear processing, functional connectivity, insula, limbic, prefrontal

## Abstract

**Background:**

Children with a history of maltreatment suffer from altered emotion processing but the neural basis of this phenomenon is unknown. This pioneering functional magnetic resonance imaging (fMRI) study investigated the effects of severe childhood maltreatment on emotion processing while controlling for psychiatric conditions, medication and substance abuse.

**Method:**

Twenty medication-naive, substance abuse-free adolescents with a history of childhood abuse, 20 psychiatric control adolescents matched on psychiatric diagnoses but with no maltreatment and 27 healthy controls underwent a fMRI emotion discrimination task comprising fearful, angry, sad happy and neutral dynamic facial expressions.

**Results:**

Maltreated participants responded faster to fearful expressions and demonstrated hyper-activation compared to healthy controls of classical fear-processing regions of ventromedial prefrontal cortex (vmPFC) and anterior cingulate cortex, which survived at a more lenient threshold relative to psychiatric controls. Functional connectivity analysis, furthermore, demonstrated reduced connectivity between left vmPFC and insula for fear in maltreated participants compared to both healthy and psychiatric controls.

**Conclusions:**

The findings show that people who have experienced childhood maltreatment have enhanced fear perception, both at the behavioural and neurofunctional levels, associated with enhanced fear-related ventromedial fronto-cingulate activation and altered functional connectivity with associated limbic regions. Furthermore, the connectivity adaptations were specific to the maltreatment rather than to the developing psychiatric conditions, whilst the functional changes were only evident at trend level when compared to psychiatric controls, suggesting a continuum. The neurofunctional hypersensitivity of fear-processing networks may be due to childhood over-exposure to fear in people who have been abused.

## Introduction

Twenty-two per cent of 11- to 17-year-olds in the UK report having experienced physical, emotional, sexual abuse or neglect by a caregiver in their lifetime (Radford *et al.* 2013) and actual maltreatment rates are likely to be even higher (Brown *et al.* 1998). Childhood maltreatment is a severe stressor that produces a cascade of physiological, neurochemical, and hormonal changes, which can lead to enduring alterations in brain structure, function and connectivity (Teicher *et al.* 2003) and is associated with negative outcomes on behavioural, emotional and social functioning (Hart & Rubia, 2012). The human brain is still developing during childhood (Sowell *et al.* 2003; de Graaf-Peters & Hadders-Algra, 2006). Hence, childhood trauma can disrupt these neurodevelopmental processes.

The ability to categorize facial expressions is generally acquired during childhood and is invaluable for social interaction. Maltreated children are exposed to atypical emotional environments, including less positive (Bugental *et al.* 1990) and more negative (Herrenkohl *et al.* 1991) emotion. Altered emotion processing is consistently reported in maltreated children, with neglected children having emotion discrimination deficits (Pollak *et al.* 2000; Fries & Pollak, 2004; Vorria *et al.* 2006) and physically abused children displaying response biases for negative emotions such as anger, fear or pain (Pollak *et al.* 2000; Pollak & Sinha, 2002; Pollak & Tolley-Schell, 2003; Pine *et al.* 2005).

Physically abused or neglected individuals, have altered event-related potential (ERP) responses when presented with angry or fearful faces, or voices, compared to happy or neutral targets (Pollak *et al.* 2001; Cicchetti & Curtis, 2005; Parker & Nelson, 2005; Shackman *et al.* 2007). Functional magnetic resonance imaging (fMRI) studies of emotion processing in neglected/institutionalized children (Maheu *et al.* 2010; Tottenham *et al.* 2011), maltreated children (McCrory *et al.* 2013) and adults with mixed maltreatment histories (Taylor *et al.* 2006; Dannlowski *et al*. 2012, 2013; Fonzo *et al*. 2013) showed enhanced activation of fronto-limbic regions, in particular the amygdala, but also hippocampus, ventromedial prefrontal cortex (vmPFC), anterior cingulate cortex (ACC) and insula, in response to negative emotions, mainly anger and fear.

Previous emotion processing fMRI studies in maltreatment have studied participants with neglect or histories of multiple forms of maltreatment. Neglected and physically maltreated participants differ in their response to emotional stimuli (Pollak *et al.* 2000) and different maltreatment types may manifest differently. For example, sexually abused and neglected children are most likely to present with social withdrawal, whereas physically abused children are most likely to have problems with aggressive/disruptive behaviour (Trickett & McBride-Chang, 1995). In adults, childhood sexual abuse is particularly associated with sexual problems, emotional abuse with low self-esteem and physical abuse with marital breakdown (Mullen *et al.* 1996). Ideally, therefore, the effects of each maltreatment type should be studied in isolation. Physical abuse was of particular interest in the current study due to the effect it is thought to have specifically on processing negative emotions (Pollak & Tolley-Schell, 2003). However, although participants were recruited based on their exposure to severe childhood physical abuse, it is unrealistic to separate physical abuse from typically co-occurring emotional abuse and neglect since they are present in almost all cases of physical maltreatment (Edwards *et al.* 2003; Trickett *et al.* 2011); hence, our maltreated group had also experienced emotional abuse and neglect. Sexual abuse was excluded due to the known differences in structural, behavioural and psychiatric consequences (Trickett & McBride-Chang, 1995; Ackerman *et al.* 1998; Heim *et al.* 2013).

The abovementioned studies have focused on regions of interest (ROIs), thereby limiting hypotheses to *a priori* regions, primarily the amygdala, providing biased, inappropriately constrained representations (Friston *et al.* 2006). Another limitation of many maltreatment studies is not controlling for typically co-occurring psychiatric conditions, rendering it impossible to determine whether observed effects are a result of the maltreatment or the associated conditions (McCrory *et al.* 2011; Hart & Rubia, 2012; Lim *et al.* 2014). In addition, all emotion processing studies have used traditional static stimuli which do not accurately represent dynamic ‘real world’ emotional expressions which rarely remain static.

Most fMRI studies of emotion processing in maltreatment have concentrated exclusively on functional activation and neglected more sophisticated functional connectivity. Functional communication between brain regions is vital in cognition and emotion, thus examination of altered functional connectivity in childhood maltreatment is vital. One study of women with post-traumatic stress disorder (PTSD) due to intimate partner violence reported that childhood maltreatment severity was correlated with limbic–prefrontal connectivity strength, specifically involving the amygdala, insula, ACC and medial frontal gyrus, while processing fearful or angry faces (Fonzo *et al.* 2013). It is important that these preliminary findings are further investigated in adolescents without secondary trauma to better understand the effect of maltreatment on brain *networks* in addition to isolated regions.

The aim of the current study was to investigate for the first time, the effect of child abuse on functional activation and connectivity in adolescents during processing of dynamic emotional facial expressions. The current study aimed to disentangle maltreatment effects from those due to psychiatric conditions by including a psychiatric control group matched with the abused group on psychiatric diagnosis. Furthermore, only medication-naive, drug abuse-free participants were included and whole-brain analyses were used. For this purpose, 20 adolescents who had experienced physical abuse, 20 psychiatric controls matched to the abused group by psychiatric diagnoses but with no abuse and 27 healthy controls were assessed during a dynamic fMRI emotion discrimination task. Based on behavioural findings of a bias towards negative emotions (Pollak *et al.* 2000; Pollak & Sinha, 2002; Pollak & Tolley-Schell, 2003) and on previous fMRI findings in neglected/institutionalized children (Maheu *et al.* 2010; Tottenham *et al.* 2011) and individuals with mixed maltreatment histories (Taylor *et al*. 2006; Dannlowski *et al*. 2012, 2013; Fonzo *et al*. 2013; McCrory *et al*. 2013), we hypothesized that maltreated individuals would demonstrate an enhanced response and show altered functional activation and connectivity of fronto-limbic networks compared to both healthy and psychiatric controls when processing negative emotions, particularly anger and fear.

## Method

### Participants

Seventy right-handed adolescents aged 12–20 participated (Table 1). Twenty-three maltreated participants were recruited through Kids Company (http://www.kidsco.org.uk/), Child and Adolescent Mental Health Services (CAMHS) and advertisements. Three were excluded due to motion, resulting in 20 participants. Maltreated participants experienced severe physical abuse prior to age 12, as defined by scores of ⩾13 on the physical abuse subscale of the Childhood Trauma Questionnaire (CTQ; Bernstein & Fink, 1998). Participants undertook the Childhood Experience of Care and Abuse (CECA) Interview (Bifulco *et al.* 1994) to ascertain detailed maltreatment histories and information was corroborated (with consent) from social services. See Supplementary Table S1A for details of onset and duration of abuse. Physically abused participants frequently had also experienced concurrent emotional abuse, emotional neglect or physical neglect (Table 1). They were diagnosed by an experienced child psychiatrist (K.A.H.M.) using the Development and Well Being Assessment (DAWBA; Goodman *et al.* 2000).

**Table 1.** Demographic, clinical and performance data for 20 maltreated adolescents, 20 psychiatric control adolescents and 27 healthy control adolescents

	Childhood maltreatment	Psychiatric controls	Healthy controls
Age, years, mean (s.d.)	17.5 (2.4)	16.8 (2.6)	17.5 (1.6)
IQ, mean (s.d.)	89.1 (12.3)	94.5 (13.2)	105.4 (10.1)
Gender, % female	30.0	50.0	29.6
% Caucasian	50.0	15.0	48.2
% Afro-Caribbean	40.0	55.0	44.4
% Mixed/other ethnicity	10.0	30.0	7.4
% No psychiatric condition	10.0	0.0	100.0
% PTSD	35.0	35.0	0.0
% PTSD with MDD	10.0	10.0	0.0
% PTSD with GAD and MDD	10.0	15.0	0.0
% PTSD with OCD	5.0	0.0	0.0
% PTSD with SP	0.0	5.0	0.0
% CD	10.0	10.0	0.0
% ODD	10.0	10.0	0.0
% Specific phobia with GAD and MDD	5.0	0.0	0.0
% PD with agoraphobia	5.0	0.0	0.0
% GAD with SP	0.0	5.0	0.0
% GAD	0.0	5.0	0.0
% PD without agoraphobia	0.0	5.0	0.0
CTQ score, mean (s.d.)			
Physical abuse	21.1 (5.0)	6.1 (1.6)	6.2 (3.4)
Sexual abuse	5.2 (0.7)	5.9 (2.0)	5.1 (0.4)
Emotional abuse	18.3 (4.2)	7.1 (1.8)	6.5 (2.6)
Emotional neglect	18.5 (4.0)	8.9 (3.8)	8.2 (3.7)
Physical neglect	14.0 (5.1)	6.8 (2.2)	6.0 (2.4)
SES score, mean (s.d.)	2.8 (0.7)	2.9 (0.7)	3.2 (0.8)
Mean reaction time, ms (s.d.)			
Neutral	610.9 (106.3)	570.3 (84.2)	661.9 (122.5)
Happy	586.3 (89.9)	578.2 (102.8)	623.8 (104.2)
Sad	653.1 (111.3)	623.7 (84.5)	716.0 (130.0)
Anger	655.5 (108.1)	632.4 (111.7)	719.3 (119.1)
Fear	657.1 (133.0)	684.7 (152.1)	738.5 (121.6)
Mean variability, ms (s.d.)			
Neutral	281.0 (40.0)	260.9 (51.2)	230.2 (32.0)
Happy	229.1 (60.8)	227.8 (52.0)	193.7 (63.1)
Sad	304.3 (52.8)	269.1 (64.7)	217.9 (60.3)
Anger	283.1 (65.7)	254.8 (49.9)	213.2 (46.3)
Fear	277.7 (59.0)	235.9 (63.3)	221.6 (48.5)
Mean % errors (s.d.)			
Neutral	16.7 (22.8)	14.8 (14.2)	10.4 (12.6)
Happy	7.2 (14.2)	11.8 (17.8)	3.1 (5.9)
Sad	18.2 (20.2)	17.5 (22.0)	10.5 (11.2)
Anger	26.3 (25.9)	16.5 (14.7)	16.8 (22.6)
Fear	19.5 (18.3)	26.7 (29.4)	17.5 (24.0)

CD, Conduct disorder; GAD, generalized anxiety disorder; MDD, major depressive disorder; ODD, oppositional defiant disorder; PD, panic disorder; PTSD, post-traumatic stress disorder; s.d., standard deviation; SP, social phobia.

Twenty psychiatric controls were recruited through Kids Company, CAMHS and advertisements. They had experienced no maltreatment (CTQ subscale scores of ⩽7 for physical abuse, ⩽8 for emotional abuse, ⩽6 for sexual abuse, ⩽9 for emotional neglect and ⩽7 for physical neglect). Diagnoses were again made using the DAWBA and matched as closely as possible one-to-one with maltreated participants (Table 1). Where psychiatric controls had PTSD, causal trauma(s) were unrelated to childhood maltreatment and included bullying, living in war-time Afghanistan, witnessing murder, car accidents and death of a loved one. See Supplementary Table S1B for details of onset and duration of trauma for the 13 participants with PTSD diagnoses.

Twenty seven healthy controls were recruited from advertisements, had experienced no maltreatment and had no psychiatric diagnoses. Both control groups were matched as closely as possible to the maltreated group in ethnicity, though for psychiatric controls matching psychiatric condition was the priority.

In addition to the CTQ and DAWBA, all participants underwent the Wechsler Abbreviated Scale of Intelligence (WASI; Wechsler, 1999) to assess IQ. Socioeconomic status (SES) was measured by two items from the Family Affluence Scale (FAS; Currie *et al.* 2008). A 10 panel T-cup urine test (http://www.testfield.co.uk) was used to test for substance abuse and participants who tested positive for any of the 10 substances were excluded (resulting in exclusion of three maltreated, one psychiatric control and one healthy control). Other exclusion criteria were left-handedness, IQ < 70, current psychoactive medication, sexual abuse (⩾6 on sexual abuse CTQ subscale), neurological disorder, major head injuries, drug and alcohol abuse, literacy problems, learning disability, psychotic illness, bipolar disorder, schizophrenia, current suicidal behaviour or general MRI contraindications. Participants received £40 as compensation for time and travel. The National Research Ethics Service approved the study and informed consent was obtained from all participants and, if below 18 years old, consent was also obtained from parents or guardians.

### fMRI emotion discrimination task

**Fig. 1.**
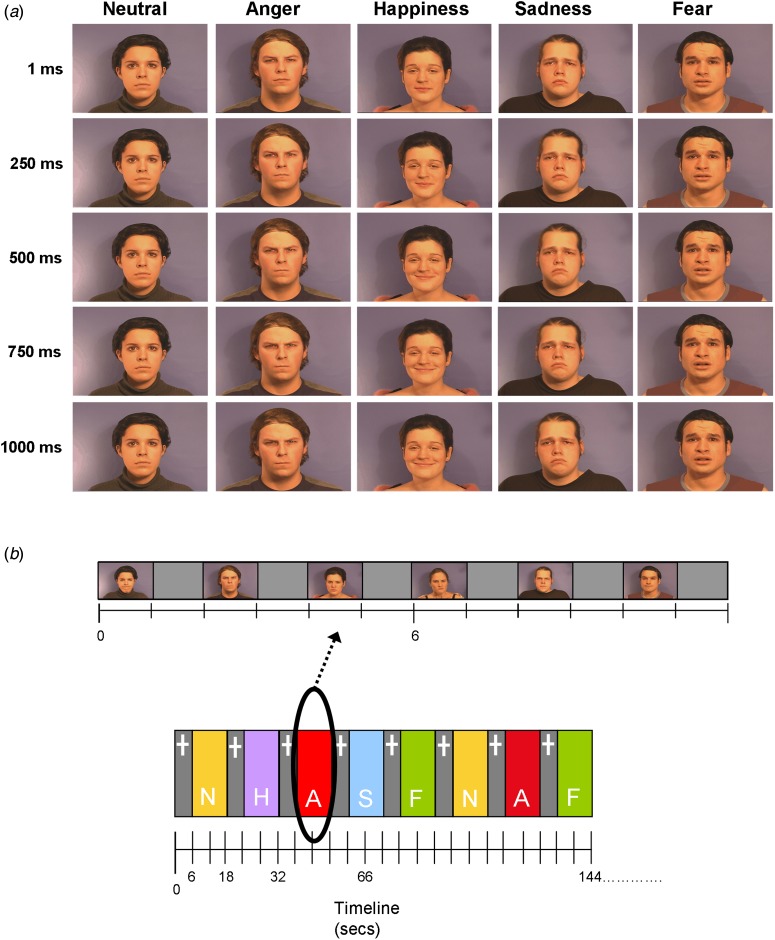
(*a*) Examples of actors expressing the five emotions: neutral, anger, happiness, sadness and fear. Five time points in the clip (1, 250, 500, 750, 1000 ms) are displayed. (*b*) Showing (top row) an example emotion block (angry) and (bottom row) the block structure of the task comprising 6-s fixation cross blocks (+) interspersed with 12-s emotion blocks (A, angry; F, fear; H, happy; N, neutral; S, sad).

Participants practiced the 8-min block design fMRI Emotion Discrimination task, which measures the ability to categorize dynamic facial expressions of emotions, once prior to scanning. Participants were shown 1-s video clips of six actors (three males) displaying neutral, fearful, angry, sad or happy expressions (Fig. 1). Clips were taken from a validated set of stimuli (Simon *et al.* 2008) and cut backward from the peak of the expression to avoid different lengths and variability of exposure. Blocks of stimuli (12 s) of each emotion were interspersed with a fixation cross baseline condition (6 s). Each emotion was presented in a block of 6 × 1-s stimuli with each stimuli followed by a 1-s gap. Each emotion block was repeated five times in a pseudo-random order and neutral was repeated six times. Participants were instructed to identify each clip as positive, neutral or negative by immediately pressing one of three buttons with the right index, middle and ring fingers, respectively.

### Performance data analysis

To test the hypothesis of enhanced responses to negative emotions in maltreated participants, *t* tests were carried out to identify group differences between each emotion in percentage errors, reaction times and response time variability. Errors were divided into negative (neutral/happy perceived as negative), positive (neutral/sad/anger/fear perceived as positive) and omission errors (no response) and *t* tests were carried out. Correlation analyses were carried out between participant age and performance variables, in order to investigate the effect of age on task performance.

### fMRI data acquisition and analysis

Data acquisition, pre-processing and first-level analysis are included in the Supplementary material. For second-level, contrast images from the first-level were used to conduct full factorial whole-brain analyses comparing activation across the three participant groups for each emotion contrasted with fixation and all negative emotions contrasted with happy. Age was entered into the analysis as a covariate as, although there were no significant group differences in age, the sample spanned a relatively wide age range. A separate exploratory analysis was also carried out which included percentage error, reaction time and response time variability as covariates. BOLD responses are reported using a cluster threshold of *p* < 0.05 family-wise error rate (FWER) corrected. Given the limited studies testing brain function differences in maltreated populations, and to control for the false positive rate (using *p* < 0.05 FWER-corrected cluster statistics) while limiting potential type II errors, we chose an a priori cluster-forming threshold of *p* < 0.01 for significant between-group differences.

MarsBar (http://marsbar.sourceforge.net/) was used to extract beta values from an 8-mm radius sphere around the peak of activation that differed between groups in order to carry out correlation analyses between neural activation and reaction time, percentage errors, response time variability (Table 1) and participant age for all participants. Additionally correlation analyses were carried out between peak beta values and maltreatment onset, duration (Supplementary Table S1A) and total CTQ scores in the maltreated group and between beta values and trauma onset and duration for psychiatric controls with PTSD (Supplementary Table S2).

### Functional connectivity analysis

As effects for fear processing were identified in the main analysis, a post-hoc functional connectivity analysis was carried out for the fear condition only. To assess functional connectivity differences between groups, a generalized psychophysiological interaction (gPPI) analysis was conducted using SPM8. A seed region in the left vmPFC was selected based on the peak of group activation differences for fear. For each participant, an average time-course was extracted from an 8-mm sphere around individual local maximum coordinates closest to the group difference peak (−6, 52, 8). Individual design matrices were computed with three regressors for each emotion, one representing the vmPFC time-course, one representing the time-dependent change as a psychological variable of interest and the third representing the element-by-element product of the previous two (the PPI term). Contrast images were generated for each subject contrasting the fear PPI term with the happy PPI term and entered into a group-level analysis. With this implementation of the gPPI analysis, significant SPM activations of a particular area would reflect changes in functional connectivity for fear processing between the left vmPFC and activated regions. Significant clusters were identified with a threshold of *p* < 0.001 uncorrected with at least 10 contiguous voxels in the cluster. The combination of height-extent thresholds effectively yields equivalent correction for multiple comparisons (Forman *et al.* 1995).

## Results

### Demographic and clinical data

Pearson's χ² tests showed no significant group differences for gender (χ^2^ = 2.494, *p* = 0.287) nor ethnicity (χ^2^ = 11.008, *p* = 0.088). One-way analyses of variance (ANOVAs) showed no significant group differences for age (*F*_2,64_ = 0.936, *p* = 0.397) nor SES (*F*_2,64_ = 2.530, *p* = 0.091) but for IQ (*F*_2,64_ = 11.817, *p* < 0.001) which is typical in this population (Carrey *et al.* 1995; De Bellis *et al.* 2009) (Table 1). *Post-hoc t* tests revealed that IQ was higher for healthy controls relative to maltreated (*p* < 0.001) and psychiatric control (*p* < 0.05) groups, who did not differ from each other (*p* = 0.735).

### Performance data

The maltreated group responded faster than healthy controls for fear (*p* < 0.05) and psychiatric controls responded faster than healthy controls for neutral (*p* < 0.01), sad (*p* < 0.05) and anger (*p* < 0.05). Maltreated and psychiatric groups had higher response time variability than healthy controls for all emotions (*p* < 0.05), but did not differ from each other except for fear where maltreated subjects had higher response time variability (*p* < 0.01). When grouped by psychiatric diagnosis rather than maltreatment and compared with those with no diagnoses, PTSD patients had higher response time variability for neutral (*p* < 0.05), sad (*p* < 0.001) and anger (*p* < 0.05); individuals with disruptive disorder diagnoses (conduct disorder/oppositional defiant disorder) had higher response time variability for neutral (*p* < 0.001) and sad (*p* < 0.05) and individuals with other anxiety disorders (generalized anxiety disorder, panic disorder, phobias) demonstrated higher response time variability for neutral (*p* < 0.001), anger (*p* < 0.001) and fear (*p* < 0.05). Individuals with other anxiety disorders had higher response time variability than those with PTSD for neutral and angry (<0.05). There were no significant differences in response time variability between participants with a disruptive disorder and those with PTSD. For neutral errors, *t* tests showed significant differences between maltreated and psychiatric groups (*p* < 0.05) and a trend for differences between maltreated and healthy controls (*p* = 0.071) where maltreated participants made more negative errors (Supplementary Table S2). No significant correlations were found between participant age and reaction time (*r* = 0.104, *p* = 0.400), percentage errors (*r* = 0.228, *p* = 0.064) nor response time variability (*r* = 0.083, *p* = 0.502).

### Motion

MANOVAs showed no significant group effects in the extent of 3-dimensional motion as measured by maximum displacement for x, y, and z axes (*F*_6,124_ = 1.043, *p* = 0.401).

### Activations and group differences for emotion conditions

**Fig. 2.**
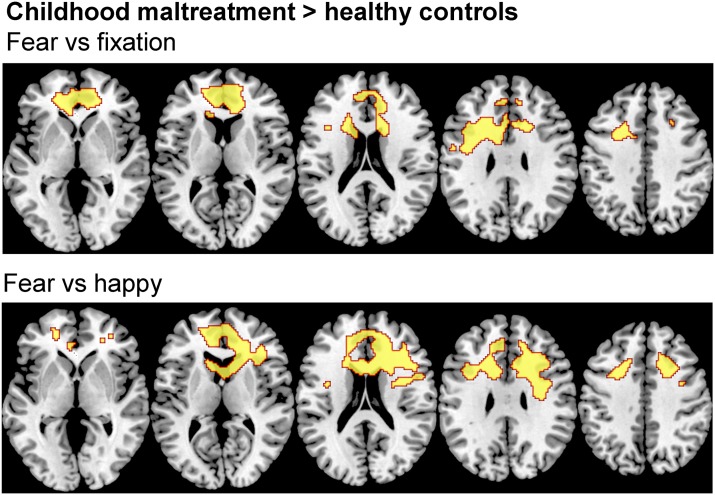
Between-group differences in brain activation for whole-brain analyses of fear *v.* happy and fear *v.* fixation contrasts. Thresholds were *p* < 0.05 family-wise error rate-corrected. *Z* coordinates represent distance from the anterior-posterior commissure in millimetres. The right side of the image corresponds to the right side of the brain.

Within-group activations for all emotions *v.* fixation are shown in Supplementary Fig. S1. Three-group ANOVAs revealed no effect of group for angry, sad or happy *v.* fixation but revealed significant group effects for fear *v.* fixation in bilateral vmPFC and ACC (*F*_2,64_ = 8.890; *p* < 0.05 FWER corrected). *Post-hoc* comparisons showed that maltreated adolescents relative to healthy controls had increased activation in a large bilateral cluster in vmPFC and ACC reaching subcortically into the caudate (Table 2, Fig. 2). Maltreated and psychiatric control groups did not differ at this threshold. To explore potential differences between these two groups, a more lenient threshold of *p* < 0.005 uncorrected was used, revealing small bilateral clusters of increased activation in occipital cortex, vmPFC and ACC (Supplementary Fig. S2).

**Table 2.** Differences in activation between physically maltreated adolescents, psychiatric control adolescents and healthy control adolescents for fear v. fixation and fear v. happy

Emotion contrast	Subject contrast	Brain regions of activation	Brodmann's area	Cluster level		Peak MNI coordinates	Voxel level *Z* value
				No. of Voxels	*p* (corr.)		
Fear *v.* fixation	CM > HC	B MFG, ACC, SFG, IFG, preCG, caudate body	6/8/9/10/24/32/47	4615	0.002	−6, 52, 8	3.93
						−34, 6, 30	3.61
						12, 52, 8	3.6
Fear *v.* happy	CM > HC	B ACC, MFG, SFG, IFG, preCG, caudate body	6/8/9/10/24/32/47	7541	<0.001	10, 18, 22	3.86
						18, 30, 20	3.82
						32, 24, 22	3.78

ACC, Anterior cingulate cortex; B, Bilateral; CM, childhood maltreatment; HC, healthy controls; IFG, inferior frontal gyrus; MFG, medial frontal gyrus; MNI, Montreal Neurological Institute; preCG, precentral gyrus; SFG, superior frontal gyrus.

*p* value is <0.05 FWER corrected.

No significant group effects were observed for angry or sad *v.* happy but there were for fear *v.* happy in a cluster comprising bilateral ACC and vmPFC reaching into the caudate (*F*_2,64_ = 8.821, *p* < 0.05 FWER corrected). *Post-hoc* comparisons showed that maltreated adolescents relative to healthy controls had increased activation in this cluster (Table 2, Fig. 2). Maltreated and psychiatric groups did not differ. At an exploratory *p* < 0.005 uncorrected threshold, maltreated adolescents demonstrated increased activation of bilateral ACC and right vmPFC compared to psychiatric controls (Supplementary Fig. S2).

A boxplot of beta values for each group from an 8-mm sphere around the peak for group activation differences for fear *v.* fixation in left vmPFC (see Supplementary Fig. S3) shows that, generally, the spread of the psychiatric controls lay somewhere in between that of the maltreated and healthy controls. An exploratory analysis was carried out including performance variables as covariates in the analysis. All main findings remained.

### Functional connectivity

**Fig. 3.**
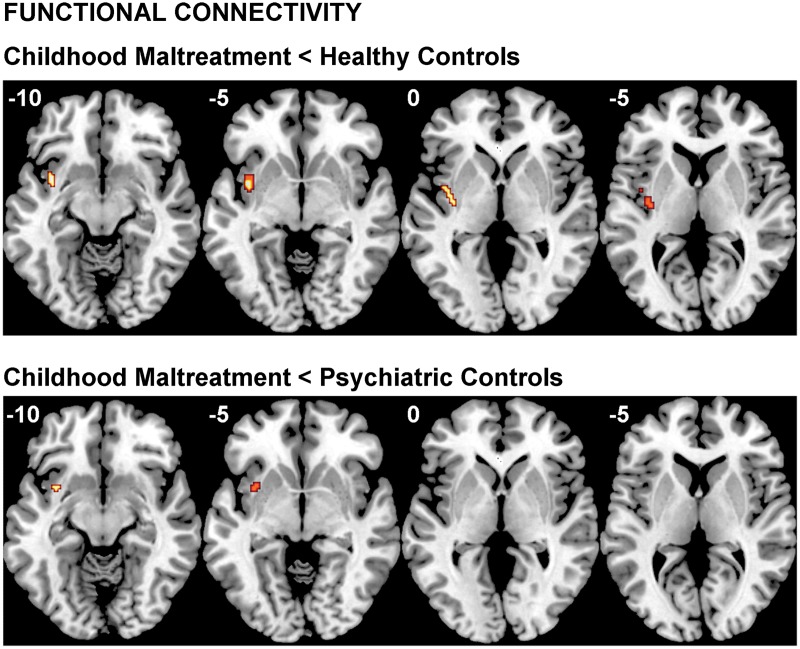
Functional connectivity group differences between the seed region of the left ventromedial prefrontal cortex and the whole brain for the fear *v.* happy and contrast. The threshold is *p* < 0.001 uncorrected with an extent threshold of 10 voxels. *Z* coordinates represent distance from the anterior-posterior commissure in millimetres. The right side of the image corresponds to the right side of the brain.

A significant group effect for connectivity was revealed between left vmPFC and left insula during fear *v.* happy (*F*_2,64_ = 8.474; *p* < 0.001). *Post-hoc* comparisons showed that maltreated adolescents relative to healthy controls had reduced connectivity between left vmPFC and a cluster in left insula and claustrum and relative to psychiatric controls in a smaller cluster in left insula (see Table 3 and Fig. 3).

**Table 3.** Differences in functional connectivity with seed region in left vmPFC between physically maltreated adolescents, psychiatric control adolescents and healthy control adolescents for fear v. happy

Emotion contrast	Subject contrast	Brain regions of altered connectivity with L vmPFC	No. of voxels	Peak MNI coordinates	*p*	*Z*
Fear *v.* happy	CM < HC	L insula/claustrum	88	−38, 4, −8	<0.001	3.67
				−36, −10, 2	<0.001	3.41
				−40, −2, 2	0.001	3.18
	CM < PC	L insula	24	−34, 8, −8	0.001	3.44

vmPFC, Ventromedial prefrontal cortex; MNI, Montreal Neurological Institute; CM, childhood maltreatment; HC, healthy controls; L, left;

Threshold is *p* < 0.001 uncorrected with a cluster extent of >10.

### Correlations

No significant correlations were found between peak beta values and reaction time (*r* = 0.145, *p* = 0.242), percentage errors (*r* = 0.155, *p* = 0.211), response time variability (*r* = 0.019, *p* = 0.878), participant age (*r* = 0.064, *p* = 0.604), onset of maltreatment (*r* = 0.204, *p* = 0.388) or trauma (*r* = 0.056, *p* = 0.849), duration of maltreatment (*r* = 0.342, *p* = 0.140) or trauma (*r* = 0.086, *p* = 0.769) nor CTQ scores (*r* = 0.042, *p* = 0.861).

## Discussion

This is the first whole-brain fMRI investigation of the effect of child abuse on functional activation and connectivity during dynamic emotion processing in adolescents. The study demonstrates that maltreated adolescents exhibited altered fear processing at behavioural and neurofunctional levels. Behaviourally, maltreated participants responded faster to fear than healthy controls. Neurofunctionally, relative to healthy controls, maltreated adolescents had increased activation in bilateral vmPFC and ACC for fear, but no differences for other emotions. They also demonstrated, albeit at a more lenient threshold, hyperactivation of ventromedial fronto-cingulate regions relative to psychiatric controls, signifying that the vmPFC/ACC effect may possibly be maltreatment-specific to a degree. Furthermore, maltreated individuals had reduced functional connectivity between left vmPFC and insula relative to both control groups, suggesting that the reduced connectivity is due to maltreatment and not associated psychopathologies Enhanced fear perception in maltreated individuals seems, therefore, to be associated with enhanced fear-related ventromedial fronto-cingulate activation and altered functional connectivity with associated limbic regions.

The behavioural hypersensitivity to fear in maltreated adolescents is consistent with previously reported negative emotion response biases (Pollak *et al.* 2000; Pollak & Sinha, 2002). The finding of hyperactivity and altered connectivity of fear processing networks is novel and extends previous findings of altered fronto-limbic activity for fear in neglected children (Maheu *et al.* 2010; Tottenham *et al.* 2011) and adults with mixed maltreatment (Taylor *et al.* 2006; Dannlowski *et al.* 2012; Fonzo *et al.* 2013) to physically and emotionally abused adolescents. We speculate that maltreated individuals respond faster to dynamic fearful expressions and show increased vmPFC/ACC activation and altered vmPFC-insular connectivity because they have experienced fear more frequently and are therefore better able to recognise fear quickly. Enhanced vmPFC/ACC activation in maltreatment in response to fear is consistent with the concept that these regions play key roles in fear processing, appraising negative emotions and regulating emotional responses via the limbic system (Phelps *et al.* 2004; Milad *et al.* 2007; Hansel & von Kanel, 2008; Etkin *et al.* 2011). The effect of childhood maltreatment on the vmPFC and ACC may reflect the fact that they are late developing, undergoing structural and functional maturation late into childhood and adolescence (Marsh *et al.* 2008; Velanova *et al.* 2008; Rubia *et al.* 2013).

Increased vmPFC/ACC activation may represent a fear regulation deficit. vmPFC and ACC are closely interconnected to limbic structures, particularly the amygdala (Amaral *et al.* 1992; Ghashghaei *et al.* 2007) and insula (Morris *et al.* 1999; Schienle *et al.* 2002; Sehlmeyer *et al.* 2009), which are both crucial for fear processing (Davis & Whalen, 2001) and top-down control of emotions (Ochsner & Gross, 2005; Etkin *et al.* 2011). The effect was much weaker when compared to psychiatric controls, which may be explained by the fact that many individuals in the psychiatric control group (the 65% with PTSD) had also experienced traumatic events, and hence may also have some degree of fear regulation deficit. Indeed, a boxplot of peak beta values for each group shows that, generally, the spread of the psychiatric controls lay between that of the maltreated and healthy controls (Supplementary Fig. S3).

Reduced top-down control over emotions is supported by the finding of diminished functional connectivity between vmPFC and insula, extending findings to the network level suggesting alteration in fear processing *networks* as well as *regions*. As mentioned above, the insula is implicated in fear processing and it is thought to convey cortical representations of fear to the amygdala (Phelps *et al.* 2001). Diminished connectivity between vmPFC and insula in maltreatment may contribute to the observed hyperactivity in ventromedial frontal regions. For example, decreased vmPFC-insula connectivity could result in weakened top-down control of vmPFC over the insula leading to a fear regulation deficit and increased fear sensitivity. Reduced functional connectivity parallels structural connectivity findings in maltreated individuals of reduced white matter tract density in the left uncinate fasciculus, which connects prefrontal with limbic regions, including amygdala and insula (Eluvathingal *et al.* 2006) and in the cingulum bundle, which connects limbic structures, including the insula, with cortical regions including the cingulate gyrus (Choi *et al.* 2009). The uncinate fasciculus and cingulum bundle both undergo development changes well into late childhood and adolescence (Lebel *et al.* 2008) and the insula is known to be involved in regulating glucocorticoids, which play a pivotal role in the stress response (Fornari *et al.* 2012).

Unexpectedly, we observed no group differences in the amygdala for fear as has been previously reported for neglected and institutionalized children (Maheu *et al.* 2010; Tottenham *et al.* 2011) and adults with mixed maltreatment (Taylor *et al.* 2006; Dannlowski *et al.* 2012). One reason could be that almost all studies that have reported functional changes in the amygdala specify the amygdala as the main, or only, ROI. Another reason could be maltreatment type differences as the neglected/institutionalized children in Maheu and Tottenham *et al.*’s studies did not have documented histories of physical maltreatment and the adults in Taylor *et al.*’s and Dannlowski *et al.*’s studies only minimal physical maltreatment. In fact, the results of the current study most resemble those from a study of women with intimate partner violence and mixed childhood maltreatment histories, including physical maltreatment (Fonzo *et al.* 2013). Fonzo and colleagues used whole-brain and limbic ROI approaches and reported that childhood maltreatment severity was correlated with ACC and insula activation and limbic-prefrontal connectivity while processing fear. An alternative explanation could be timing. The hippocampus is fully developed by age 2, amygdala volume peaks at around age 10, but the PFC matures relatively late in life with progressive volumetric changes into adulthood and a sharp growth between 8 and 14 years of age (Lupien *et al.* 2009). Because of its protracted development, the PFC is conceivably more vulnerable to environmental stressors such as child abuse than are the limbic structures.

Also surprising was lack of correlation between blood oxygen-level dependent (BOLD) beta values and abuse onset, duration and severity (CTQ scores). Previous work has shown relationships between CTQ scores and BOLD signal during face processing (Edmiston & Blackford, 2013). A possible explanation is that the current study only tested potential relationships between BOLD signal and maltreatment measures for the maltreated group who had all experienced severe childhood abuse, whereas Edmiston & Blackford studied a group of young adults with an inhibited temperament with larger variability in degrees of maltreatment, which might be better suited to finding such correlations.

The perception of neutral expressions as negative by maltreated individuals may stem from hyper-vigilance to negative emotions as has been reported in depression (Oliveira *et al.* 2013; Maniglio *et al.* 2014) and social anxiety (Cooney *et al.* 2006). Many of the maltreated group were diagnosed with depression and anxiety so it is unsurprising that they have a tendency to misattribute neutral faces as negative but it is difficult to rationalise the fact that this phenomenon was not observed in the psychiatric controls, who had extremely similar depression and anxiety diagnoses. The current study reported higher response time variability for both maltreated and psychiatric control groups, relative to healthy controls. As this was present for both maltreated and psychiatric groups it is possible that this was related to specific psychiatric condition(s). However, no clear relationship was evident between response time variability and diagnosis.

Among the strengths of this study are that all participants were medication-naive and drug-free, and their abuse experience was carefully assessed and corroborated by social services. Also, we included a psychiatric control group to determine the specificity of maltreatment in our findings. The whole-brain approach ensured that effects outside the expected ROIs were not missed. Finally, the use of dynamic stimuli which more accurately mimic the way in which emotional expressions are observed in everyday life is a novelty of the study.

Limitations include that we cannot categorically state that effects are a result of exclusively physical abuse as many participants also experienced neglect and emotional abuse. However, separating physical from emotional abuse and neglect is unrealistic as the vast majority of maltreated children are subjected to more than one abuse type, with less than 5% occurring in isolation (Ney *et al.* 1994). Ideally we would like to have investigated specific effects of emotional and physical abuse on neural responses but unfortunately physical and emotional abuse were highly correlated in the sample (*r* = 0.922, *p* < 0.001). IQ was not matched between groups which could be considered a limitation. However, since lower IQ is associated with childhood maltreatment (Carrey *et al.* 1995; De Bellis *et al.* 2009), artificially matching groups on IQ is inappropriate as it creates unrepresentative groups and it is misguided to covary for a pre-existing group difference as this would lead to potentially spurious results (Miller & Chapman, 2001; Dennis *et al.* 2009). Another limitation is the inclusion of mixed genders as maltreatment may affect the genders differently (Cooke & Weathington, 2014). Finally, for nine out of 20 participants, maltreatment continued beyond age 12 (Supplementary Table S1A). The broad range of developmental ages at which the maltreatment ended could have affected the neurodevelopmental changes observed.

## Conclusion

Childhood abuse is associated with faster fear processing, which was concomitant with elevated activation of vmPFC and ACC and decreased connectivity between vmPFC and insula. Findings suggest that maltreatment leads to behavioural and neurofunctional sensitisation to fearful expressions through altered activation and connectivity of fear processing networks. These alterations could have negative consequences for socio-emotional interaction and this knowledge may help develop new interventions to address social information errors.

## Supplementary material

For supplementary material accompanying this paper visit http://dx.doi.org/10.1017/S0033291716003585. click here to view supplementary material
