# Supplementary material for: Altered fear processing in adolescents with a history of severe childhood maltreatment: an fMRI study
Source: Psychol Med. 2018 Feb 12;48(7):1092–101. doi: 10.1017/S0033291716003585 (PMC6088776; doi:10.1017/S0033291716003585)
Supplement: Supplementary file 1 [file S0033291716003585sup001.doc]

**SUPPLEMENTARY MATERIAL**

***fMRI Data Acquisition***

Gradient-echo echoplanar MR imaging (EPI) data were acquired on a GE SIGNA HDx 3T system at the Centre for Neuroimaging Sciences, King’s College London. A semi-automated quality control procedure ensured consistent image quality (Simmons *et al*., 1999). The body coil was used for RF transmission and an 8 channel headcoil for reception. In each of 23 non-contiguous planes parallel to the anterior-posterior commissure, 237 interleaved T2*-weighted MR images depicting BOLD (Blood Oxygen Level Dependent) contrast covering the whole brain were acquired with TE=40ms, TR=2s, flip angle=75°, in-plane resolution=3mm, slice thickness=5mm (slice-skip=0.5mm). A high-resolution gradient EPI was also acquired in the inter-commissural plane, with TE=30ms, TR=1.8s, flip angle=90°, 43 slices, slice thickness=3.0mm, slice skip=0.3mm, 1.875mm in-plane voxel size (matrix size 128x128), providing complete brain coverage.

***fMRI Pre-processing and 1^st^ level anlaysis***

Data were analysed using SPM8 (www.fil.ion.ucl.ac.uk/spm). Images were motion corrected with all images being realigned to the first scan in the time-series and then the mean image. After realignment, images were co-registered to the high resolution EPI. All scans were normalised to standard space, using the EPI template, with the parameters derived from the high resolution EPI and applied to the functional time series. Data were spatially smoothed using a kernel of 8mm full-width half-maximum.

At the first level, a general linear model (GLM) approach was followed to allow for contrasts of each emotion condition against fixation. Regressors were convolved with the canonical hemodynamic response function to model the BOLD (blood oxygen level dependent) signal. Regressors for events of interest were fixation, neutral, fearful, angry, sad and happy. First- and second-order movement parameters were also included in the model as regressors for events of no interest. A high-pass filter (128 seconds) was applied to the data and first-order temporal autocorrelation was modelled. Weighted contrasts were used to test the effect of changes on BOLD signal during each emotion contrasted with fixation and all negative emotions contrasted with happy for each individual. Given that the neutral condition was twice as likely to be perceived as negative by the maltreated group (Supplementary Table S2), all negative emotion conditions were contrasted with happy rather than neutral. Contrasting negative emotions with happy is common practice in fMRI studies of emotion processing (McClure *et al*., 2007;Whalen *et al*., 2008;Klumpp *et al*., 2012). Furthermore, the neutral condition did not contain the same amount of facial movement as the emotion conditions and hence the happy condition is a better matched contrast for the negative emotion conditions as it controlled for motion perception.

***Supplementary Table S1:***

**A)** Information regarding onset and duration of abuse (in years) for the 20 maltreated participants (* denotes participants for whom abuse continued after 12 years of age)

| **Participant** | **Age at Onset** | **Duration** |
| --- | --- | --- |
| *S007 | 6 | 13 |
| *S008 | 4 | 11 |
| *S010 | 5 | 8 |
| S013 | 0 | 9 |
| S015 | 0 | 11 |
| S016 | 0 | 12 |
| S017 | 0 | 6 |
| *S020 | 5 | 11 |
| *S022 | 10 | 4 |
| *S025 | 3 | 12 |
| S029 | 4 | 6 |
| S030 | 6 | 6 |
| S031 | 5 | 6 |
| *S032 | 5 | 11 |
| *S034 | 6 | 11 |
| S035 | 2 | 5 |
| S036 | 2 | 7 |
| S050 | 3 | 7 |
| *S055 | 3 | 12 |
| S057 | 8 | 3 |

**B)** Information regarding trauma type, onset and duration (in years) for the 13 psychiatric controls with PTSD

| **Participant** | **Trauma** | **Age at Onset** | **Duration** |
| --- | --- | --- | --- |
| S026 | Peer bullying | 5 | 6 |
| S027 | Afghanistan war | 1 | 14 |
| S028 | Afghanistan war | 1 | 13 |
| S033 | Peer bullying | 5 | 8 |
| S037 | Car accident & injury | 11 | 2 |
| S041 | Witnessed death & bullying | 8 | 5 |
| S043 | Mugged/Assaulted | 15 | 0.5 |
| S045 | Death of loved one | 12 | 0.1 |
| S048 | Death of loved one | 12 | 0.1 |
| S059 | Witnessed death | 16 | 0.1 |
| S066 | Afghanistan war | 2 | 12 |
| S069 | Witnessed attack | 14 | 0.1 |
| S070 | Peer bullying | 7 | 7 |

***Supplementary Table S2:*** Breakdown of behavioural errors into negative, positive, neutral and omission errors for the three groups for all emotion conditions as mean % error (standard deviation).

|  | **Childhood Maltreatment** | | | | **Psychiatric Control** | | | | **Healthy Control** | | | |
| --- | --- | --- | --- | --- | --- | --- | --- | --- | --- | --- | --- | --- |
| **Emotion** | **Positive** | **Negative** | **Neutral** | **Omission** | **Positive** | **Negative** | **Neutral** | **Omission** | **Positive** | **Negative** | **Neutral** | **Omission** |
| **Neutral** | 4.9 (7.5) | 6.0 (13.9) | / | 5.8 (6.5) | 2.5 (4.5) | 2.5 (4.1) | / | 9.0 (13.1) | 3.0 (6.5) | 3.2 (5.9) | / | 4.2 (4.6) |
| **Happy** | / | 1.3 (4.5) | 2.2 (6.8) | 3.5 (5.0) | / | 1.8 (2.8) | 1.2 (2.0) | 8.2 (17.0) | / | 0.1 (0.6) | 0.7 (1.9) | 1.6 (4.3) |
| **Sad** | 2.0 (6.7) | / | 10.5 (14.3) | 5.5 (5.2) | 1.0 (2.2) | / | 7.5 (14.8) | 8.3 (12.0) | 0.1 (0.6) | / | 6.2 (9.5) | 4.0 (5.) |
| **Anger** | 3.5 (8.7) | / | 13.5 (17.6) | 8.3 (8.8) | 2.8 (3.3) | / | 3.8 (5.2) | 9.2 (13.0) | 1.2 (2.6) | / | 11.1 (20.1) | 3.6 (8.1) |
| **Fear** | 1.7 (7.5) | / | 9.7 (12.3) | 8.0 (6.5) | 1.5 (3.5) | / | 12.8 (27.2) | 10.7 (13.0) | 1.4 (3.7) | / | 9.6 (18.5) | 4.3 (5.4) |

***Supplementary Figure S1:*** One-sample T-tests for each emotion condition vs fixation reported at FWER corrected cluster level threshold p<0.05 overlaid onto an axial template image (HC= healthy controls, PC= psychiatric controls, CM= childhood maltreatment). Z-coordinates represent distance from the anterior–posterior commissure in mm. The right side of the image corresponds to the right side of the brain.

***
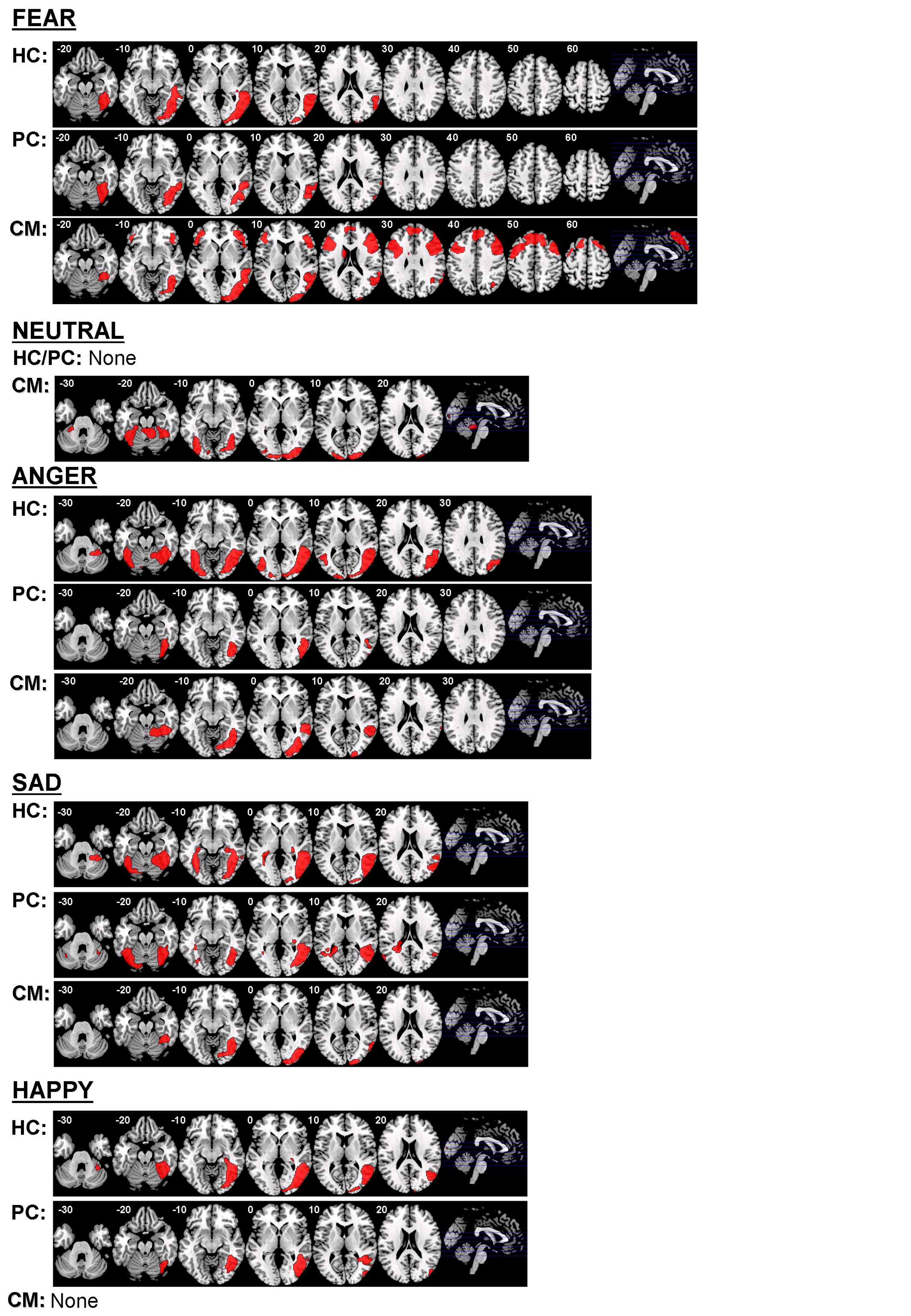
***

**Supplementary Figure S2:** Between group differences in brain activation for whole brain analyses of fear vs happy and fear vs fixation contrasts for childhood maltreatment > psychiatric control comparisons. These contrasts are presented at a lenient exploratory threshold of P < 0.005 uncorrected. Z-coordinates represent distance from the anterior–posterior commissure in mm. The right side of the image corresponds to the right side of the brain.

**
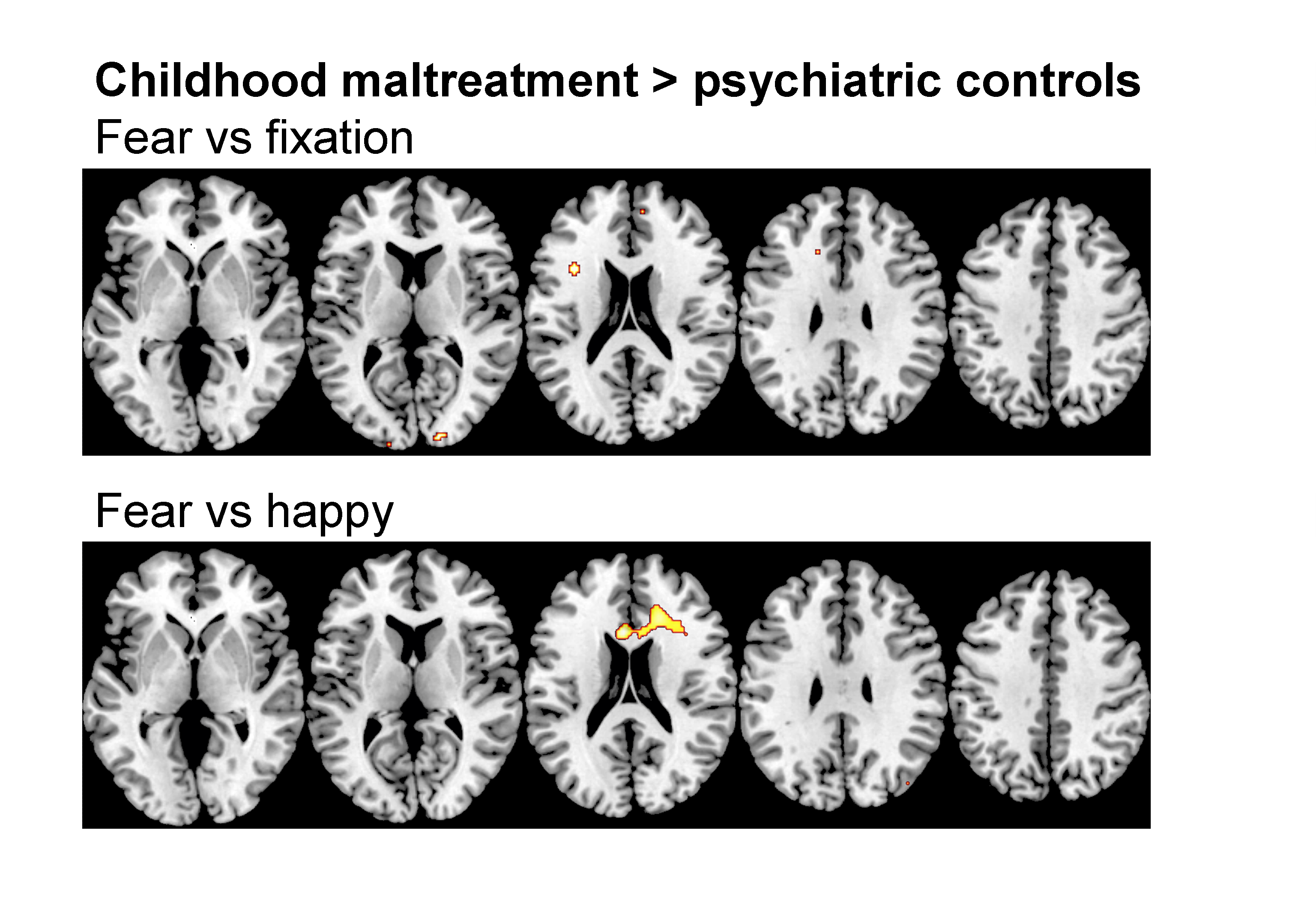
**

**Supplementary Figure S3:** A boxplot of beta values for each group from an 8mm sphere around the peak voxel for group activation differences in the left vmPFC.


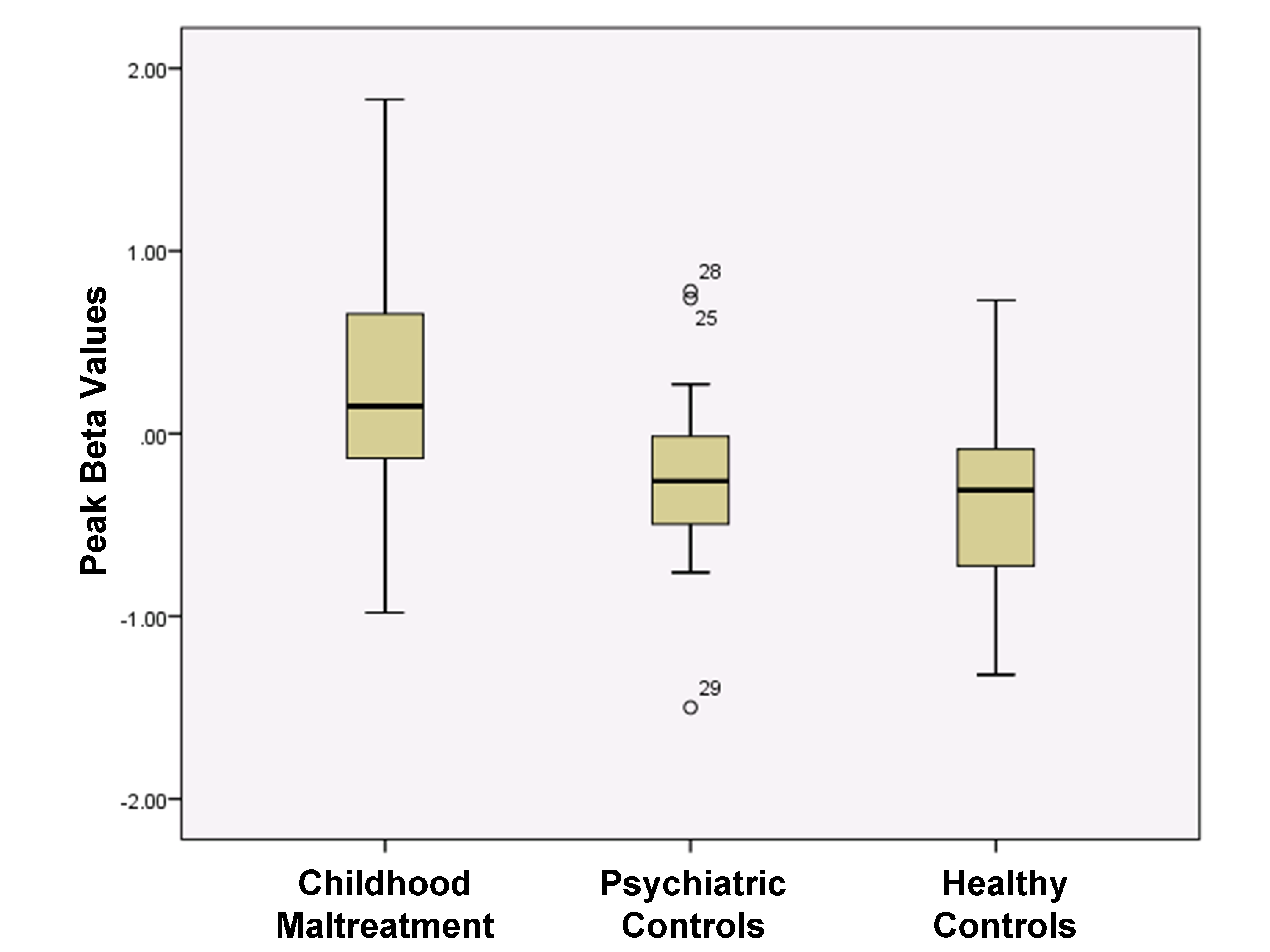


**References**

**Klumpp, H., Angstadt, M. & Phan, K. L.** (2012). Insula reactivity and connectivity to anterior cingulate cortex when processing threat in generalized social anxiety disorder. *Biological Psychology* **89**, 273-276.

**McClure, E. B., Adler, A., Monk, C. S., Cameron, J., Smith, S., Nelson, E. E., Leibenluft, E., Ernst, M. & Pine, D. S.** (2007). fMRI predictors of treatment outcome in pediatric anxiety disorders. *Psychopharmacology* **191**, 97-105.

**Simmons, A., Moore, E., Williams, S.C.R.** (1999). Quality Control for Functional Magnetic Resonance Imaging Using Automated Data Analysis and Shewhart Charting. *Magnetic Resonance in Medicine* **41**, 1274-1278.

**Whalen, P. J., Johnstone, T., Somerville, L. H., Nitschke, J. B., Polis, S., Alexander, A. L., Davidson, R. J. & Kalin, N. H.** (2008). A functional magnetic resonance imaging predictor of treatment response to venlafaxine in generalized anxiety disorder. *Biological Psychiatry* **63**, 858-863.
